# Effects of SGLT2 Inhibitors on Cardiac Mechanics in Hispanic and Black Diabetic Patients

**DOI:** 10.3390/jcm13154555

**Published:** 2024-08-04

**Authors:** Errol Moras, Rishi Shrivastav, Kruti D. Gandhi, Dhrubajyoti Bandyopadhyay, Ameesh Isath, Akshay Goel, Jonathan N. Bella, Johanna Contreras

**Affiliations:** 1 Department of Medicine, Mount Sinai Morningside-West Hospitals, Icahn School of Medicine at Mount Sinai, New York, NY 10025, USA; 2 Division of Cardiology, Bluhm Cardiovascular Institute, Northwestern University Feinberg School of Medicine, Chicago, IL 60611, USA; 3 Division of Cardiology, Massachusetts General Hospital, Harvard Medical School, Boston, MA 02114, USA; 4 Division of Cardiology, Westchester Medical Center, Valhalla, NY 10595, USA; 5 Division of Cardiology, BronxCare Hospital, Bronx, NY 10457, USA; 6 Division of Cardiology, The Mount Sinai Hospital, Icahn School of Medicine at Mount Sinai, New York, NY 10029, USA

**Keywords:** SGLT2 inhibitors, Blacks, Hispanics, echocardiography, global longitudinal strain

## Abstract

**Background:** Clinical trials demonstrating improved cardiovascular outcomes with SGLT2 inhibitors have often had limited representation from Black and Hispanic populations. While the mechanisms of action are not well known, ethnicity- or gender-based receptor physiology may render SGLT2 inhibitors a better agent in certain populations over others. **Methods:** A medical records query yielded diabetic patients initiated on SGLT2 inhibitors between 2013 and 2020. Patients with coronary artery disease, cardiac arrhythmias, and heart failure were excluded. Transthoracic echocardiographic studies (TTE) before and after starting SGLT2 inhibitors were analyzed, and post-processing left ventricular global longitudinal strain (LV GLS) analysis was also performed on each echocardiographic study. Univariate outliers and patients with missing data were excluded. **Results:** Among 94 patients with TTE (mean age 60.7 years; 68% Hispanics, 22.3% Blacks; median follow up of 7 months), there were significant improvements in the mean LV GLS (−15.3 vs. −16.5; *p* = 0.01), LV mass (LVM) (198.4 ± 59.6 g vs. 187.05 ± 50.6 g; *p* = 0.04), and LV mass index (LVMI) (100.6 ± 26.6 g/m^2^ vs. 94.3 ± 25.4 g/m^2^; *p* = 0.03) before and after initiating SGLT2 inhibitors but no significant change in the ratio (MV E/E’) of peak early diastolic mitral flow velocity (E) and spectral pulsed-wave Doppler-derived early diastolic velocity from the septal mitral annulus (E’) (12.5 ± 5.7 vs. 12.7 ± 4.8; *p* = 0.38). Changes in HbA1c (r^2^ = 0.82; *p* = 0.026), LVM (r^2^ = 0.20; *p* = 0.04), and LVMI (r^2^ = 0.20; *p* = 0.04) were found to be independently associated with changes in values of LV GLS on follow-up echocardiograms, when compared to the pre-medication LV GLS number. **Conclusion:** Non-White diabetic patients receiving SGLT2 inhibitors against a backdrop of other cardioprotective medications demonstrate significant improvements in LV remodeling and LV GLS, driven in part by an improvement in glycemic control. Large, prospective studies are needed to explore the differences in the therapeutic actions of SGLT2 inhibitors among different populations.

## 1. Introduction

Cardiovascular diseases represent a substantial comorbidity among individuals diagnosed with type 2 diabetes (T2DM), affecting nearly 18% of patients concurrently with coronary artery disease and approximately 9–22% of patients with comorbid heart failure (HF) [1]. In the United States, the prevalence of T2DM is the highest among Hispanic and Blacks, compared to non-Hispanic Whites [2]. Sodium-glucose cotransporter-2 (SGLT2) inhibitors represent a novel drug class that inhibits glucose reabsorption in the proximal convoluted tubule of the nephron by blocking the sodium/glucose co-transporter-2 protein [3]. This induces glycosuria and natriuresis, effectively regulating blood glucose levels. Robust evidence from large, randomized control trials, such as the CANVAS program, DECLARE-TIMI 58, and EMPA-REG OUTCOME, demonstrates significant improvements in renal and cardiovascular outcomes among diabetic patients with SGLT2 inhibitor use [4,5,6]. In a pre-specified meta-analysis of patients with chronic heart failure from five randomized clinical trials—DAPA-HF, EMPEROR-Reduced, SOLOIST-WHF, DELIVER, and EMPEROR-Preserved—SGLT2 inhibitors were found to significantly reduce the risk of mortality and worsening heart failure, as well as improve patient symptoms and overall health status across the full spectrum of the ejection fraction [7,8,9,10,11,12]. The 2022 guidelines from the American Heart Association (AHA)/American College of Cardiology (ACC)/Heart Failure Society of America (HFSA) now include a Class I recommendation to initiate SGLT2 inhibitors for preventing heart failure-related hospitalization and cardiovascular death in patients with heart failure with a reduced ejection fraction (HFrEF). They also include a Class IIa recommendation for patients with heart failure with a mildly reduced ejection fraction (HFmrEF) and heart failure with a preserved ejection fraction (HFpEF) [13]. However, outcomes from these large trials were not uniformly studied across minority subgroups, such as Black or Hispanic populations, who constituted only 4–7% of the enrolled patients. 

Traditionally renal sodium-handling mechanisms have shown notable variations based on ethnicity in individuals from specific ethnic backgrounds, such as those of Black ancestry showing an increased predisposition to volume retention [14]. This phenomenon suggests a preference for specific classes of medications among patients based on their ethnicity. Prior studies reveal that despite higher rates of death and HF hospitalizations, patients of Black ethnicity are less likely to receive established guideline-directed medical therapy than their Caucasian counterparts [15]. The potential benefits of SGLT2 inhibitor medications in reducing HF hospitalizations may be linked to their favorable effects on left ventricular (LV) function [7,8]. Left ventricular global longitudinal strain (LV GLS), studied by two-dimensional speckle-tracking echocardiography, provides a more accurate evaluation of contractile function and allows for the early detection of subtle dysfunctions, compared to LVEF [16,17]. To date, few clinical studies have explored the effects of SGLT2 inhibitors on cardiac mechanics, particularly LV functional parameters [18,19]. This literature gap is especially pronounced in Hispanic and Black populations, which exhibit the highest rates of cardiovascular complications among all diabetic patient populations [20]. Therefore, we conducted a study to investigate the effects of SGLT2 inhibitors on the parameters of LV cardiac mechanics in Black and Hispanic diabetic populations. 

## 2. Methods

### 2.1. Study Population

Our study was a single-center retrospective investigation that enrolled diabetic patients initiated on SGLT2 inhibitors who were referred to the echocardiography lab of a hospital in New York City between 2013 and 2020 for the evaluation of signs and symptoms indicative of cardiovascular etiology. 

### 2.2. Search Strategy, Inclusion, and Exclusion Criteria

We utilized the electronic medical record system to identify diabetic patients initiated on SGLT2 inhibitors with available echocardiographic studies conducted before the initiation of medication. The follow-up echocardiogram was defined as the one performed closest to the commencement of medication. The inclusion criteria of our study were (1) age > 18 years; (2) diabetic patients who were newly initiated on SGLT2 inhibitors; (3) patients admitted for the evaluation of symptoms and signs suggestive of cardiovascular etiology; (4) availability of echocardiographic studies performed before and after the initiation of SGLT2 inhibitors; and (5) adequate quality of echocardiographic data that are amenable to speckle-tracking analysis for the calculation of GLS. Patients were excluded if they met any of the following criteria: (1) age < 18 years; (2) evidence of any terminal disease; (3) evidence of underlying cardiovascular conditions, such as heart failure with a reduced ejection fraction, coronary artery disease (documented through a positive cardiovascular stress test or abnormal coronary angiogram), atrial fibrillation, ventricular tachycardia, ventricular fibrillation, or more than mild valvular heart disease, at the initiation of medications; (4) poor echocardiographic images not suitable for post-processing LV GLS; (5) no echocardiographic studies available within 12 months prior to the initiation of SGLT2 inhibitors; and (6) patients with a renal estimated glomerular filtration rate (eGFR) < 45 mL/min/m^2^ at the time of medication initiation. The study received approval from the institutional ethics committee.

### 2.3. Echocardiographic Data

Both sets of echocardiographic studies for each patient were conducted at rest using commercially available echocardiographic systems (GE Vivid E95 and Vivid Q, Horten, Norway; Philips EPIQ7, Andover, MA, USA). The echocardiograms with the calculation of GLS were repeated for 3–12 months after the initiation of SGLT2 inhibitor therapy. Digital routine grayscale two-dimensional cine loops from three consecutive heartbeats were obtained via standard parasternal and apical views. Gain settings and sector width were adjusted to optimize endocardial definition and ensure complete myocardial visualization, respectively. All standard echocardiographic parameters were measured following the guidelines from the American Society of Echocardiography [21]. Subsequently, all images were uploaded to a central server in the Digital Imaging and Communications in Medicine (DICOM) format and made available for offline analysis by a dedicated cardiology fellow or attending physician. Investigators reading and reporting the echocardiograms were blinded to all aspects of the patient’s clinical and demographic information, including the date and time the study was performed.

To calculate the LVEF, apical two- and four-chamber views were utilized employing the modified biplane Simpson’s method. The ratio (MV E/E’) of the trans-mitral velocity obtained via pulse-wave Doppler imaging (E) and the early diastolic mitral septal annular velocity obtained by spectral tissue Doppler imaging (E’) was used to estimate the filling pressure of the left ventricle. Following this, speckle-tracking strain analysis was performed using commercially available software (TomTec-Arena, TomTec Imaging Systems, GmbH, Unterschleißheim, Germany) to calculate the left ventricular global longitudinal strain (LV GLS). Apical four-chamber, two-chamber, and long-axis cine loop images in DICOM format were used for each echocardiographic study to calculate post-processing LV GLS. 

**Figure 1:**
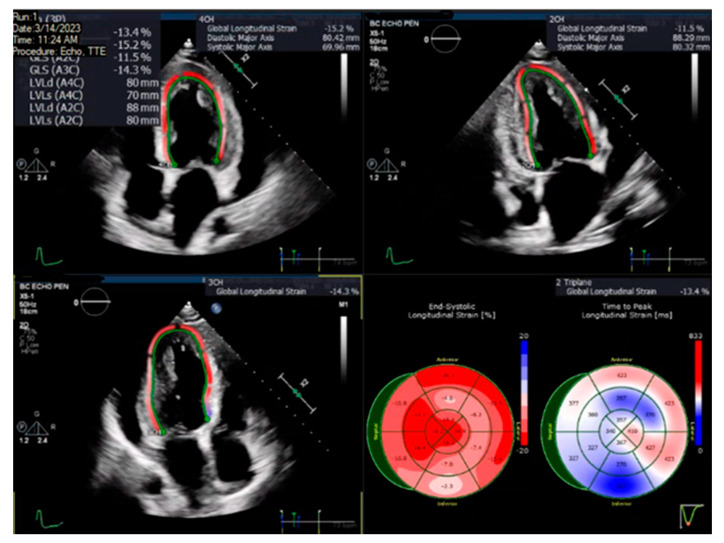
Image showing two-dimensional speckle-tracking analysis depicted in color-coded segments to form a representative bullseye plot to estimate the left ventricular global longitudinal strain in a patient.

A pre-specified contour-detecting algorithm built into the software was utilized to estimate the longitudinal speckle-tracking strain. If deemed necessary by the analyzing physician, the contour was adjusted to incorporate the best region of interest. Strain results were visualized using color-coded segments on a 16-segment model of the left ventricle, and the GLS value was calculated as the averaged longitudinal strain of all segments, expressed as an absolute value. Figure 1 illustrates an example of the LV GLS calculated in one of the patients.

### 2.4. Statistical Analysis

Continuous variables for normally distributed data were expressed as mean ± SD, whereas categorical variables were expressed as frequencies and percentages. The normality for data was assessed using the Kolmogorov–Smirnov test. Paired *t*-tests and repeated analysis of variance (ANOVA) were carried out to compare the differences in variables between baseline and post-SGLT2 inhibitor groups. Differences between categorical variables were analyzed using the Chi-square test and Fisher’s exact test. Independent associations of changes in the echocardiographic parameters between baseline and follow-up echocardiograms, namely LV mass (LVM), left ventricular filling pressures (as estimated by the ratio of E/E’), and LV GLS, to baseline demographic and clinical variables were evaluated by multiple regression analysis. Differences were deemed statistically significant if *p* < 0.05. All analyses were performed using commercially available software (SPSS software version 24.0, SPSS Inc., Chicago, IL, USA).

## 3. Results

The initial search yielded 359 patients, of whom 185 were excluded due to the presence of one or more cardiovascular conditions, 44 were excluded due to the absence of either pre- or post-procedure echocardiograms, and an additional 32 were excluded due to poor echocardiographic data deemed not amenable to the application of speckle-tracking analysis for the calculation of GLS. Out of the remaining 98 patients, 4 were considered outliers based on their standardized z-scores and were subsequently excluded from the study, resulting in a final sample size of 94. Among the 94 participants that were included in the final analysis, 64 (68.1%) were Hispanic, and 21 (22.3%) were Black. The baseline clinical, laboratory, and echocardiographic characteristics of the enrolled patients are presented in Table 1. The mean age of the participants was 60.7 ± 9 years, with 73 (77.7%) being females. Mean baseline blood pressure was 131/77 mmHg, and hemoglobin A1c (HbA1c) was 8.3%. The mean LVEF on pre-medication echocardiograms was 65.9 ± 6.6%, and the median time between the initiation of medication and the follow-up echocardiogram was 7 months. The mean ages for Black and Hispanic patients were 58.4 years and 62.1 years, respectively, and the mean durations from the initiation of SGLT2 inhibitors to the follow-up echocardiogram were not significantly different between the two groups (7.5 months for Blacks vs. 6.1 months for Hispanics; *p* = 0.19). No difference was noted in the baseline characteristics of Black and Hispanic patients. However, Black patients demonstrated a statistically significant improvement in the HbA1c at follow-up (8.8% to 7.9%; *p* = 0.04), whereas Hispanic patients failed to show an improvement (8.2% to 7.9%; *p* = 0.2). 

**Figure 2:**
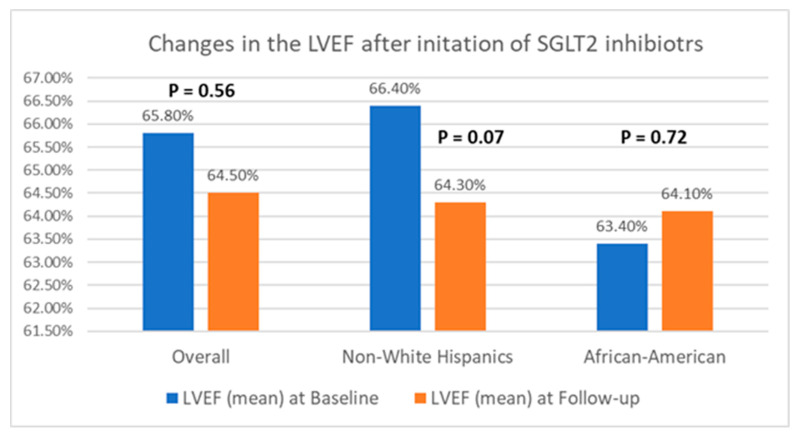
Graphical representation of changes in LVEF after the initiation of SGLT2 inhibitors.

**Figure 3:**
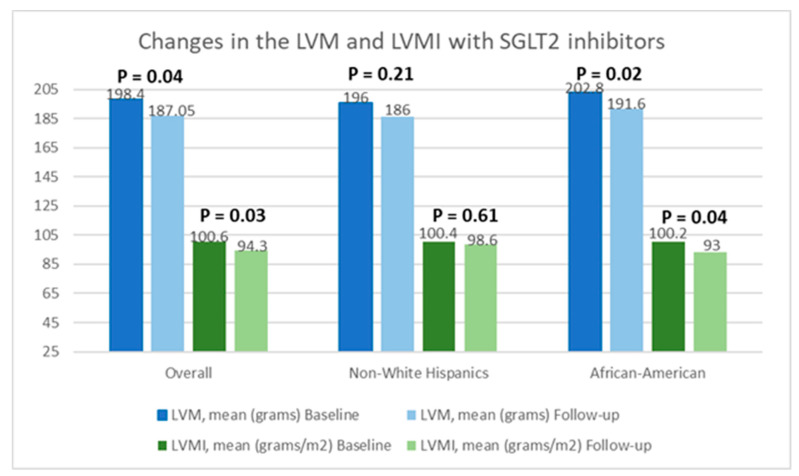
Graphical representation of changes in the left ventricular mass (LVM) and left ventricular mass index (LVMI) after the initiation of SGLT2 inhibitors.

**Figure 4:**
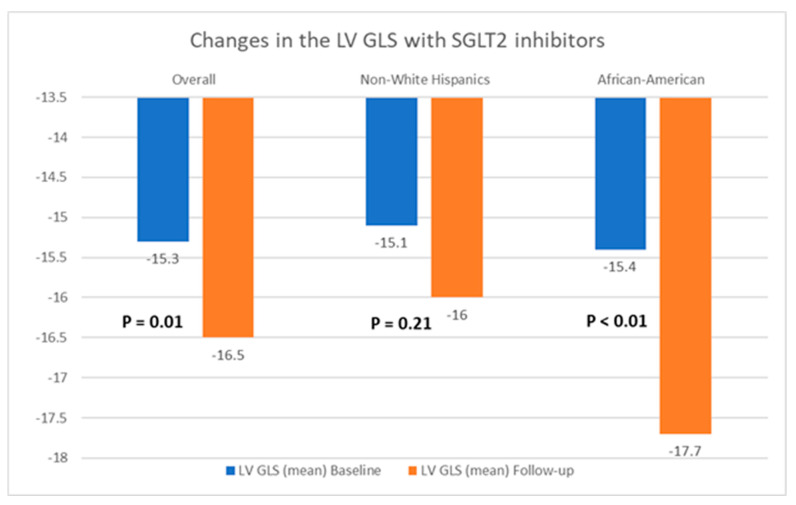
Graphical representation of changes in the left ventricular global longitudinal strain (LV GLS) after the initiation of SGLT2 inhibitors.

Table 2 presents a comparison of echocardiographic variables measured at baseline and following the initiation of SGLT2 inhibitors in the overall population, delineating observed changes and their statistical significance in response to treatment. Although no significant difference was noted in the left ventricular ejection fraction (LVEF) before and after SGLT2 inhibitor administration, statistically significant reductions in the mean LVM (198.4 ± 59.6 g vs. 187.05 ± 50.6 g; *p* = 0.04) and mean LVMI (100.6 ± 26.6 g/m^2^ vs. 94.3 ± 25.4 g/m^2^; *p* = 0.03) were observed, compared to baseline. Additionally, there was an improvement in the mean LV GLS from −15.3 ± 3.1% to −16.5 ± 3.1% (*p* = 0.01). However, the mean left ventricular filling pressures, as estimated by MV E/E’, did not show any significant change, compared to baseline (12.5 ± 5.7 vs. 12.7 ± 4.8; *p* = 0.38). Further, a stratified analysis based on patients’ ethnicity was conducted to evaluate changes in echocardiographic parameters in response to medication. Among Hispanics, no significant differences were observed in any of the measured echocardiographic parameters pre- and post-SGLT2 inhibitors administration. However, among Black patients, except for mean LVEF (63.4 ± 8.2% vs. 64.1 ± 9.3%; *p* = 0.72) and MV E/E’ (11.8 ± 3.4 vs. 12.4 ± 3.8; *p* = 0.60), significant reductions were noted in mean LVM (202.8 ± 57.8 g vs. 191.6 ± 66.7 g; *p* = 0.02), mean LVMI (100.2 ± 28.9 g/m^2^ vs. 93.0 ± 30.5 g/m^2^), and mean LV GLS (−15.4 ± 3.5% vs. −17.7 ± 2.7%; *p* < 0.01) following the initiation of SGLT2 inhibitors. The noteworthy alterations in select echocardiographic parameters, evaluated across both the entire population and diverse ethnic patient subgroups, are graphically depicted in Figure 2, Figure 3 and Figure 4.

Table 3 shows the results of the multiple regression analysis for the association of changes in LV GLS values with various demographic and clinical variables. Changes in HbA1c (r^2^ = 0.82; *p* = 0.026), LVM (r^2^ = 0.20; *p* = 0.04), and LVMI (r^2^ = 0.20; *p* = 0.04) were found to be independently associated with changes in values of LV GLS on follow-up echocardiograms, when compared to the pre-medication LV GLS number. Similarly, LV GLS was the only variable shown to be statistically significantly associated with changes in LVM (r^2^ = 1.345; *p* = 0.037). No other significant association was found for either of these two parameters with various independent demographic, clinical, and echocardiographic variables. 

## 4. Discussion

Our retrospective analysis of echocardiograms, conducted predominantly on non-White diabetic patients before and after the initiation of SGLT2 inhibitors, revealed statistically significant improvements in LVM and LVMI, along with an enhanced LV GLS, on the follow-up echocardiogram. A subsequent analysis based on patients’ ethnicity failed to demonstrate significant changes in the echocardiographic parameters among Hispanic patients. However, the usage of SGLT2 inhibitors did prevent any further worsening of cardiac remodeling in this group. In contrast, among Black patients, we observed a significant reverse cardiac remodeling characterized by improvements in LVM, LVMI, and LV GLS following the initiation of SGLT2 inhibitors. 

Data from clinical trials have demonstrated substantial improvements in heart failure (HF) hospitalization and cardiovascular mortality among heart failure patients treated with SGLT2 inhibitors, irrespective of their diabetes status [8,9]. While the precise mechanism underlying the cardioprotective effects of SGLT2 inhibitors remains under investigation, several theoretical frameworks have been proposed to elucidate their modes of action. Some of the suggested mechanisms include their natriuretic and antihypertensive effects, improved glycemic control, enhanced myocardial energetics, calcium homeostasis, altered adipokine regulation, impact on oxidative stress modulation, myocardial extracellular matrix remodeling, and lower systemic inflammatory response [22,23]. Given their higher renal sodium reabsorption and lower urinary sodium excretion, Black patients are predisposed to a higher incidence of heart failure [14]. For similar reasons, Morris et al. argued that SGLT2 inhibitors might indeed exert a more favorable therapeutic effect in Black patients, compared to patients of other ethnicities [24]. Although this pathophysiology is common among many patients, it is particularly significant for Black individuals, who exhibit the highest prevalence of salt sensitivity (up to 75% of Black individuals with hypertension) and the highest prevalence of heart failure. Numerous previous studies have highlighted the significant challenge of healthcare disparities among Black and Hispanic populations, compared to their White counterparts [25,26]. Wang et al.’s analysis of the Medical Expenditure Panel Survey (MEPS) (1996–2001) found a significantly lower rate of new prescription drug utilization among Blacks, compared to Whites [15]. Similarly, a retrospective analysis of medical and pharmacy claims data by McCoy et al. showed that among all diabetic patients, those of Black ethnicity with pre-existing cardiovascular and kidney diseases were less likely to be prescribed SGLT2 inhibitors [27].

Multiple studies have previously demonstrated the adverse cardiovascular outcomes linked to increased LVM and LVMI in a varied population, irrespective of the presence of cardiovascular conditions [28,29]. Similarly, the presence of increased LVM in diabetic patients without a history of prior cardiac diseases is independently associated with a higher incidence of future cardiovascular events [30]. LV GLS, a novel echocardiographic parameter providing crucial information on LV systolic function not readily apparent from LVEF assessment, has been shown to possess a superior prognostic value in detecting subtle echocardiographic changes in patients [31,32]. Consequently, we opted to utilize these parameters to evaluate the amelioration of or improvement in left ventricular remodeling in our study. In line with the findings of Kosugi et al. and Gamaza-Chulián et al., our analysis also identified significant improvements in LVM, LVMI, and LV GLS following the administration of SGLT2 inhibitors in diabetic patients without significant cardiac disease [33,34]. In a study conducted by Leung et al., among patients with poorly controlled type 2 diabetes, improved glycemic control over 12 months significantly enhanced LV systolic and diastolic functions, with the greatest benefits observed in those with the largest HbA1c reductions and the lowest HbA1c levels at follow-up. Moreover, improvements in systolic function and HbA1c were associated with improved filling parameters and exercise tolerance, as reflected by the reduction in the E/e′ ratio [35]. However, in contrast to our study, information on ethnicity was either missing, or the patient sample did not sufficiently represent traditionally underrepresented backgrounds. Similar to our findings, the EMPA-HEART CardioLink-6 trial reported a substantial reduction in LVMI among patients with type 2 diabetes mellitus on Empagliflozin at a 6-month follow-up [36]. However, participants included in this trial exhibited evidence of coronary artery disease. In concordance with our assessment, the EMPA-HEART trial demonstrated a swift and sustained improvement in LV GLS among diabetic patients exhibiting subclinical myocardial dysfunction, defined as LV-GLS < −16.5%, but without evidence of overt heart disease [37]. It is noteworthy that several studies have identified evidence of subclinical LV dysfunction based on LV GLS values in asymptomatic diabetic patients with normal LVEF, a phenomenon also prominently observed in our analysis [38,39]. Similar to our findings, Tanaka et al. reported a significant improvement in LV GLS values after six months of Dapagliflozin therapy; however, it is pertinent to note that the patients included in their analysis had a diagnosis of heart failure [40]. Data from the echocardiography-based correlation of LV mass and geometry with LV GLS in patients with and without diabetes have consistently demonstrated a deterioration in strain as left ventricular remodeling or hypertrophy progresses, a trend also mirrored in our findings [41,42]. The current literature investigating changes in LV filling pressures in response to SGLT2 inhibitors, as assessed by the ratio MV E/E’, presents conflicting findings. Notably, most studies reporting an enhancement in diastolic filling parameters primarily included patients diagnosed with heart failure [40,43]. In alignment with our assessment, Gamaza-Chulián et al. also observed no improvement in the E/E’ ratio, whereas the IDDA trial demonstrated a significant enhancement in the diastolic reserve of diabetic patients without heart failure or coronary artery disease who were treated with Dapagliflozin [34,44]. The absence of a significant change in our study may be attributed to the relatively small sample size. 

The EMPA-TROPISM (ATRU-4) trial, incorporating a substantial proportion of Black and Hispanic non-diabetic patients with heart failure and reduced ejection fraction (constituting up to 69% of the total participants), observed a notable improvement in cardiac magnetic resonance imaging (cMRI)-based parameters of LV remodeling and functional indices in patients treated with Dapagliflozin, compared to those on the placebo [45]. Despite considerable differences in study participants and methodologies compared to our own, the trial’s findings of improved parameters of LV remodeling align with our results. Notably, Dochert et al.’s post hoc analysis of the DAPA-HF trial, comparing the efficacy of Dapagliflozin in Black versus White patients, revealed a similar reduction in the risk of heart failure hospitalization and cardiovascular death across both populations, even though Black patients constituted only 15% of all participants [46]. However, the authors concluded that, due to a higher risk of worsening outcomes, Black patients derived a greater overall benefit in reducing the risk of adverse cardiovascular outcomes from the use of Dapagliflozin, compared to White patients. Similarly, we observed a significant improvement in echocardiographic parameters of LV remodeling in Black patients, despite a relatively small proportion. However, this, in part, could be attributed to a significant improvement in glycemic control, as evident in our univariate and multivariate analyses, a finding which has been independently shown to improve LV remodeling [35]. These findings underscore the need for further research into the precise mechanism of action of SGLT2 inhibitors, particularly in the context of varying physiology based on ethnicity.

### Study Limitations

While our study stands as the inaugural examination of the impact of SGLT2 inhibitors on echocardiographic variables of cardiac remodeling in traditionally underrepresented populations with diabetes, it is not without limitations. First, the smaller sample size of our study and the relatively short-term follow-up period may render it statistically underpowered to accurately estimate the overall impact of SGLT2 inhibitors on each echocardiographic variable. Second, there was a lack of incorporation of crucial echocardiographic parameters like LV and left atrial (LA) volumes, which could have demonstrated impacts on the outcomes in our study. Third, the absence of a control group limits our ability to draw definitive conclusions about the effects of SGLT2 inhibitors compared to standard care. Fourth, we did not account for interobserver, intraobserver, and test–retest variability and reproducibility of measurements for the echocardiographic parameters, since this was beyond the goal of our study. Lastly, the generalizability of our findings, specifically the GLS parameters, can be restricted to the vendor-specific software and imaging equipment used in our study.

## 5. Conclusions

SGLT2 inhibitors used concurrently with other cardioprotective medications promotes improvements in the left ventricular mass and left ventricular global longitudinal strain in non-White diabetic patients, as seen at the population level. Large, prospective studies are needed to explore the differences in therapeutic actions of SGLT2 inhibitors among different populations and at the individual level. 

## Figures and Tables

**Table 1:** Baseline and follow-up clinical, echocardiographic, and laboratory characteristics of diabetic patients initiated on SGLT2 inhibitors.

Characteristics	Total N = 94 (%)	Non-White Hispanics N = 64 (%)	African American N = 21 (%)	*p*-Value
Age, mean (SD) (years)	60.7 ± 9	62.1 ± 9.7	58.4 ± 8.6	0.13
Females	73 (77.7)			
Age Group (years)				
<50	10 (10.6)	6 (9.4)	2 (9.5)	
51–60	35 (37.2)	21 (32.8)	11 (52.4)	
61–70	34 (36.2)	25 (39.1)	5 (23.8)	
>70	15 (16)	12 (18.7)	3 (14.3)	
Ethnicity				
Hispanics	64 (68.1)			
African American	21 (22.3)			
Non-Hispanic Whites	4 (4.3)			
Others	5 (5.3)			
SBP, mean (SD) (mm Hg)	131.1 ± 18.7	130.3 ± 19.7	136.7 ± 15.8	0.13
DBP, mean (SD) (mm Hg)	77.4 ± 8.1	78.5 ± 7.3	80.2 ± 7.8	0.56
Body mass index, mean (SD) (kg/m^2^)	34.9 ± 7.6	34.1 ± 7.2	37.5 ± 8.3	0.11
HbA1c, mean (SD) (%)	8.3 ± 1.7	8.2 ± 1.6	8.8 ± 1.9	0.21
Follow-up HbA1c, mean (SD) (%)		7.9 ± 1.5	7.9 ± 1.4	0.89
Pro-BNP (pg/mL), mean (SD)		105.4 ± 106.2	86.3 ± 101.9	0.26
Follow up period, mean (SD) (months)		6.1 ± 7.2	7.5 ± 4.2	0.19
Age <75 years	86.65 (78.9)			
Hypertension	88 (93.6)	61 (95.3)	19 (90.5)	0.59
Hyperlipidemia	88 (93.6)	58 (90.6)	18 (85.8)	
Obesity	63 (67.0)	36 (56.3)	12 (57.1)	
Active Smoking	35 (37.2)	22 (34.4)	9 (42.8)	0.14
Substance use	15 (16.0)			
Cocaine/Amphetamine	7 (7.4)	5 (7.2)	2 (9.5)	0.53
Opioid/Methadone	4 (4.2)			
Cannabis	4 (4.2)			
Medications at the time of initiation of SGLT2 inhibitor therapy				
Metformin	71 (75.5)	50 (78.1)	14 (66.7)	0.38
Insulin	46 (48.9)	29 (45.3)	12 (57.1)	0.86
DPP-4 inhibitors	28 (29.7)	20 (31.2)	5 (23.8)	0.51
GLP-1 agonists	18 (19.1)	11 (17.2)	5 (23.9)	0.52
Sulfonylureas	12 (12.7)	7 (10.9)	4 (19.0)	0.33
Beta blockers	42 (44.7)	34 (53.1)	12 (57.1)	0.11
ACE inhibitors	50 (53.2)	36 (56.2)	11 (52.4)	0.75
ARB	16 (17.0)	11 (17.2)	4 (19.0)	0.84
CCB	42 (44.6)	32 (50.0)	10 (47.7)	0.83
Statins	81 (86.1)			
SGLT2 inhibitors				
Empagliflozin	46 (48.9)			
Canagliflozin	43 (45.8)			
Dapagliflozin	5 (5.3)			
Medications added during follow-up				
Beta blockers	16 (17.0)	10 (15.6)	3 (14.3)	0.58
ACE inhibitors	5 (5.4)	4 (6.2)	1 (4.7)	0.80
ARB	19 (20.2)	3 (4.7)	1 (4.7)	0.98
CCB	20 (21.3)	12 (18.7)	5 (23.8)	0.61

SGLT2: sodium-glucose cotransporter-2; SD: standard deviation; SBP: systolic blood pressure; DBP: diastolic blood pressure; HbA1c: hemoglobin A1c; BNP: brain natriuretic peptide; DPP: dipeptidyl peptidase 4; GLP: glucagon-like peptide; ACE: angiotensin-converting enzyme; ARB: angiotensin receptor blocker; CCB: calcium channel blockers; LVM: left ventricular mass; LVMI: left ventricular mass index; MV: mitral valve; E: peak early diastolic mitral flow velocity; E′: spectral pulsed-wave Doppler-derived early diastolic velocity from the septal mitral annulus; GLS: global longitudinal strain measures from speckle-tracking analysis.

**Table 2:** Echocardiographic variables before and after the initiation of SGLT2 inhibitors.

Overall				Non-White Hispanics			African American		
Parameter	Baseline	Follow-Up	*p*-Value	Baseline	Follow Up	*p*-Value	Baseline	Follow-Up	*p*-Value
LVEF, mean (SD) (%)	65.8 ± 5.9	64.5 ± 8.2	0.56	66.4 ± 5.8	64.3 ± 8.5	0.07	63.4 ± 8.2	64.1 ± 9.3	0.72
LVM, mean (SD) (g)	198.4 ± 59.6	187.05 ± 50.6	0.04	196.0 ± 60.3	186.0 ± 45.6	0.21	202.8 ± 57.8	191.6 ± 66.7	0.02
LVMI, mean (SD) (g/m^2^)	100.6 ± 26.6	94.3 ± 25.4	0.03	100.4 ± 26.6	98.6 ± 24.3	0.61	100.2 ± 28.9	93.0 ± 30.5	0.04
MV E/E’, mean (SD)	12.5 ± 5.7	12.7 ± 4.8	0.38	12.9 ± 6.5	13.1 ± 5.3	0.81	11.8 ± 3.4	12.4 ± 3.8	0.60
LV GLS (%), mean (SD)	−15.3 ± 3.1	−16.5 ± 3.1	0.01	−15.1 ± 3.0	−16.0 ± 5.4	0.21	−15.4 ± 3.5	−17.7 ± 2.7	<0.01

SGLT2: sodium-glucose cotransporter-2; SD: standard deviation; LVEF: left ventricular ejection fraction; LVM: left ventricular mass; LVMI: left ventricular mass index; MV: mitral valve; E: peak early diastolic mitral flow velocity; E′: spectral pulsed-wave Doppler-derived early diastolic velocity from the septal mitral annulus; GLS: global longitudinal strain measures from speckle-tracking analysis.

**Table 3:** Multiple regression analysis for the association of the change in the left ventricular global longitudinal strain (ΔLV GLS) and the change in the left ventricular mass (ΔLVM).

Independent Variable	Change in Left Ventricular Global Longitudinal Strain (ΔLV GLS)				Change in Left Ventricular Mass (ΔLVM).			
	Coefficient	Standard Error	T-Value	*p* -Value	Coefficient	Standard Error	T-Value	*p* -Value
Age	0.081	0.069	1.167	0.247	−0.116	0.742	−0.157	0.876
Gender	−0.946	1.374	−0.688	0.493	-18.247	14.734	−1.238	0.219
BMI	0.012	0.085	−0.146	0.884	−1.222	0.917	−1.333	0.186
SBP	0.003	0.043	−0.077	0.939	−0.131	0.457	−0.288	0.774
DBP	0.106	0.094	−1.124	0.264	0.107	1.012	0.106	0.916
HTN	0.862	2.681	−0.322	0.749	20.223	28.759	0.703	0.484
Smoking	0.428	1.211	−0.353	0.725	3.005	12.986	−0.231	0.818
Pro-BNP	0.006	0.006	0.936	0.352	0.014	0.051	0.271	0.787
Change in HbA1c	0.828	0.364	2.273	0.026	0.020	2.816	0.007	0.994
Beta-blockers	−0.066	1.195	−0.055	0.956	−8.805	10.037	0.877	0.383
ACE inhibitor	−0.189	1.395	0.136	0.892	−14.048	11.669	−1.204	0.232
ARB	−0.558	1.768	−0.315	0.753	−0.246	14.881	0.017	0.987
Metformin	−0.165	1.405	−0.118	0.907	−3.650	15.064	−0.242	0.809
Insulin	−0.714	1.355	0.527	0.600	−4.318	14.536	−0.297	0.767
GLP-1 agonist	−0.996	1.625	0.613	0.542	−0.885	17.429	0.051	0.960
LVEF	0.107	0.091	1.176	0.243	−0.424	0.770	−0.550	0.584
LVM	0.205	0.022	0.955	0.041				
LVMI	0.337	0.050	0.550	0.020				
MV E/E’ ratio	0.018	0.109	0.167	0.868	0.185	0.914	0.202	0.841
LV GLS					1.345	1.571	0.856	0.037

BMI: body mass index; SBP: systolic blood pressure; DBP: diastolic blood pressure; HTN: hypertension; BNP: brain natriuretic peptide; HbA1c: hemoglobin A1c; ACE: angiotensin-converting enzyme; ARB: angiotensin receptor blocker; GLP: glucagon-like peptide; LVEF: left ventricular ejection fraction; LVM: left ventricular mass; LVMI: left ventricular mass index; MV: mitral valve; E: peak early diastolic mitral flow velocity; E′: spectral pulsed-wave Doppler-derived early diastolic velocity from the septal mitral annulus.

## Data Availability

The data cannot be shared due to confidentiality protection of the patients.
